# Structured reporting of dental panoramic images in a hospital-based radiology setting: a comparative study

**DOI:** 10.3389/fdmed.2026.1769864

**Published:** 2026-03-05

**Authors:** Moritz Ludwig Schnitzer, Gloria Biechele, Emilia Schober, Felix L. Herr, Christian Dascalescu, Maurice Heimer, Ricarda Ebner, Victoria Fusch, Sebastian Marschner, Stefanie Corradini, Florian Georg Ortner, Matthias Frank Frölich, Fabian Baier, Tobias Graf, Johannes Rübenthaler, Thomas Geyer

**Affiliations:** 1 Department of Radiology, University Hospital Munich, Ludwig Maximilian University of Munich, Munich, Germany; 2 Department of Radiation Oncology, University Hospital, Ludwig Maximilian University of Munich, Munich, Germany; 3 Department of Radiation Oncology, Universitätsklinikum Erlangen, Friedrich-Alexander-Universität Erlangen-Nürnberg (FAU), Erlangen, Germany; 4 Department of Oral and Maxillofacial Surgery and Facial Plastic Surgery, University Hospital LMU Munich, Munich, Germany; 5 Department of Radiology and Nuclear Medicine, University Medical Center Mannheim, Heidelberg University, Mannheim, Germany; 6 Department for Radiotherapy, University Hospital Regensburg, Regensburg, Germany; 7 Department of Prosthetic Dentistry, University Hospital, LMU Munich, Munich, Germany

**Keywords:** clinical documentation, dental diagnostics, dental panoramic images, dental panoramic imaging, free-text reports, structured reporting

## Abstract

**Background:**

Despite radiological advancements, free-text reporting remains common. Structured reporting (SR) improves completeness, clarity, communication, efficiency, and information extraction while benefiting education and research. Its success depends on well-designed, modality-specific templates.

**Methods:**

This study aimed to compare SRs and narrative reports (NRs) for dental panoramic images in a hospital-based radiology department with respect to completeness, clarity, ease of information extraction, and clinical utility.Fifty dental panoramic images were randomly selected from the clinical archive of a tertiary-care hospital radiology department. NRs by radiologists were compared with SRs retrospectively created by a board-certified dentist using a decision-tree template (Smart Radiology platform). A questionnaire, developed with two additional dentists, assessed completeness, clarity, ease of information extraction, and clinical utility.

**Results:**

SRs outperformed NRs in all areas. Clinical questions were answered in 100% of SRs vs. 42% of NRs (*p* < 0.05). SRs enabled clinical decision-making in 100% of cases vs. 29% for NRs (*p* < 0.05). Key features were omitted in 3% of SRs but in 96% of NRs (*p* < 0.05). Information extraction was rated “easy/fast” in 100% of SRs vs. 52% of NRs (*p* < 0.05). Evaluators showed greater trust in SRs (Likert score: 5.97 vs. 4.27, *p* < 0.05).

**Conclusions:**

SR significantly enhances report quality, clinical decision-making, and communication in dental panoramic imaging.

## 1 Introduction

Dental panoramic imaging (DPI) is a widely used tomographic imaging technique in dental and maxillofacial diagnostics, offering a panoramic view of the dentition, jaws, and adjacent structures (1–3). It provides a layer thickness of 9–20 mm, capturing the maxilla, mandible, temporomandibular joints, and surrounding anatomy. Proper patient positioning and adherence to imaging guidelines are essential for diagnostic accuracy, preventing misinterpretations and unnecessary radiation exposure. However, DPI is prone to artifacts such as motion blur, distorted projections from foreign objects, and superimpositions with anatomical structures like the hyoid bone or cervical spine (1, 3).

The main advantages of DPI include its ability to provide a comprehensive overview of dental and maxillofacial structures, aiding in the detection of unexpected pathologies. It is efficient, requires minimal patient cooperation, and involves a relatively low radiation dose compared to intraoral radiographs, which provide higher resolution and are superior in the detection of tooth decay (4, 5). However, DPI has limitations, including lower resolution, geometric distortion, and difficulties in detecting early-stage pathologies. Additionally, overlapping structures, particularly in the premolar region, can reduce diagnostic clarity (6, 7).

Despite its widespread use, DPI has limitations in imaging frontal teeth and assessing the temporomandibular joints, as it does not visualize the articular disc, similar to many other radiographic and tomographic modalities, which also lack the soft-tissue contrast required for disc evaluation (8). Nevertheless, it remains the gold standard for dentomaxillofacial imaging and plays a crucial role in diagnosing and planning treatments for conditions such as periodontal disease, temporomandibular joint disorders, and pretherapeutic assessments. These include the exclusion of dental infections prior to organ transplantation, oncological therapies (e.g., chemotherapy, radiotherapy), or medication regimens (e.g., bisphosphonates). The clinical utility of DPI, however, strongly depends on the quality and clarity of radiological reporting (7, 9).

Traditionally, radiological findings have been documented in a free-text format (NRs), allowing for flexible and individualized descriptions of imaging features. While this approach enables personalization and adaptation to complex cases, it is prone to inconsistencies, variability in terminology, and omission of critical information, potentially leading to misinterpretations and delayed decision-making (10–14). In contrast, structured reporting (SR) has emerged as a standardized alternative, providing predefined templates with guided sections, decision trees, and terminology to enhance clarity, completeness, and reproducibility of radiological assessments. SR has been increasingly implemented in various imaging modalities, demonstrating improvements in report quality, communication between healthcare providers, and clinical decision-making (15–22). However, its application in dental radiology, particularly for DPI, remains largely unexplored.

The present study aims to evaluate the impact of SR compared to NR in DPI interpretation. By assessing the completeness, clarity, and clinical utility of SR vs. NR, this study seeks to determine whether SR could enhance diagnostic accuracy, could facilitate information extraction, and could improve interdisciplinary communication. Additionally, the study will examine the potential influence of SR on workflow efficiency and the overall quality of radiological reporting in oral and maxillofacial imaging. The findings will contribute to the ongoing discussion on the benefits and limitations of SR and its applicability in dental and maxillofacial radiology.

## 2 Materials and methods

### 2.1 Study design

**Figure 1:**
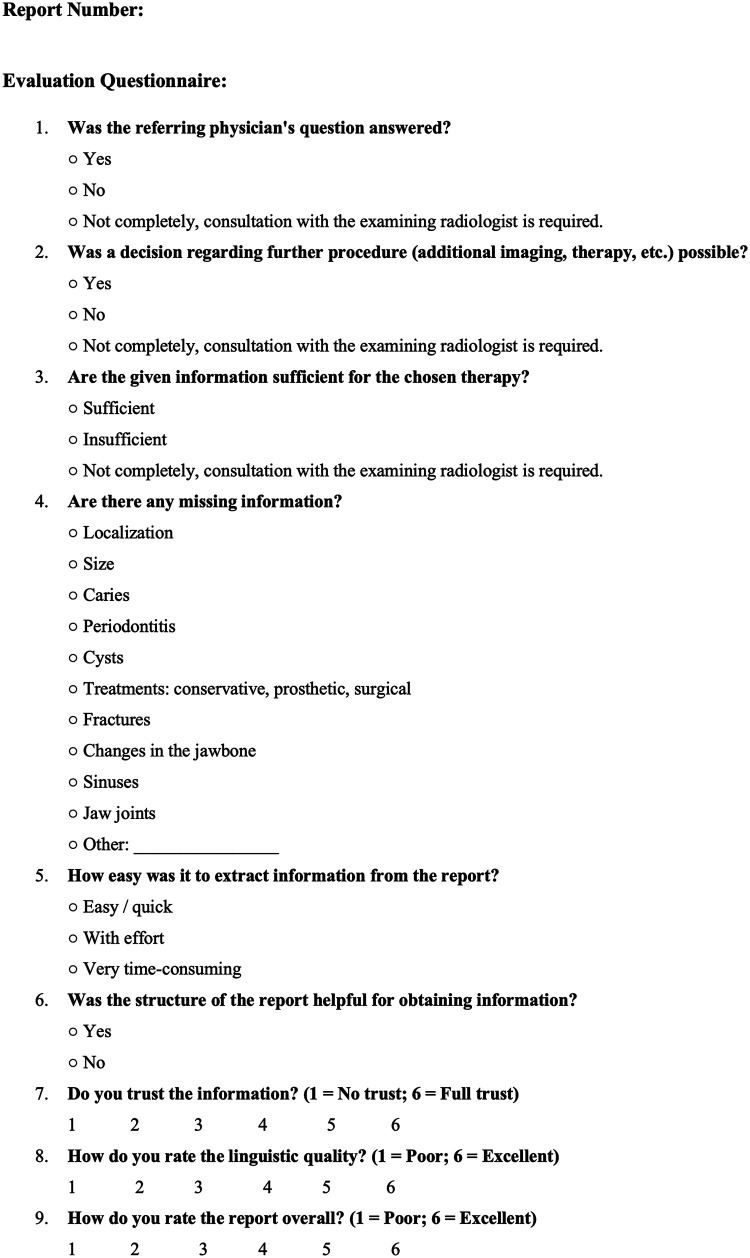
Questionnaire for the evaluation of NR and SR evaluated by two board-certified dentists.

This retrospective study was conducted at a department of Radiology. NRs from 50 randomly selected DPIs, spanning a period of over 13 years (December 2008—February 2022), and authored by various medical radiologists, were analyzed. Because this was an exploratory study, no formal *a priori* sample size calculation was performed. A sample of 50 dental panoramic images was chosen based on feasibility and in line with sample sizes used in previous structured reporting studies, yielding a total of 100 reports (50 narrative and 50 structured) for evaluation (23–25). The study aimed to evaluate the potential benefits of SR for DPIs by creating SRs for the 50 selected DPIs using a template generated with the Smart Radiology platform. These SRs were then compared to the existing NRs using a custom-designed questionnaire, which was evaluated by two independent, board-certified dentists (Figure 1). Because the narrative reports were authored by radiologists with varied clinical backgrounds and the SRs were created by a board-certified dentist, the comparison inherently reflects differences in professional training in addition to differences in reporting format.

This retrospective study was approved by the Ethics Committee and adheres to ethical guidelines for publications in the field of diagnostics (Ethics Committee of LMU Munich, approval no. 23-0172). All collected data comply with the principles of the Declaration of Helsinki.

### 2.2 Data collection and acquisition

#### 2.2.1 Image acquisition

DPIs were acquired over a 13-year period (December 2008–February 2022) using the Sirona Orthophos XG 3D Ready device. Reports were authored by various radiologists, and 50 randomly selected DPIs were retrieved from the clinical archive via the RIS Nexus/Chili and PACS Visage Imaging systems.

The selected DPIs covered diverse clinical indications, categorized into four groups: 29 (58%) for pre-transplantation listing, 8 (16%) for planned medication (e.g., bisphosphonates), 5 (10%) prior to oncologic therapies (e.g., radiotherapy or chemotherapy), and 8 (16%) for other indications, such as focus search in cases of paravalvular abscesses near an aortic valve.

#### 2.2.2 Generation of free-text reports

The NRs for the 50 DPIs, obtained from the radiological information system Nexus/Chili, were created between December 2008 and February 2022 by various radiologists in daily clinical practice. These reports were generated using a speech recognition software system without predefined text modules or structured templates [Philips SpeechMagic 6.1, Build 543 SP1 (7/2007), Philips Speech Recognition Systems GmbH, Vienna, Austria]. All NRs underwent final validation by board-certified radiologists, ensuring that they were supervised, approved, and archived by a qualified radiologist.

#### 2.2.3 Generation of structured reports

**Figure 2:**
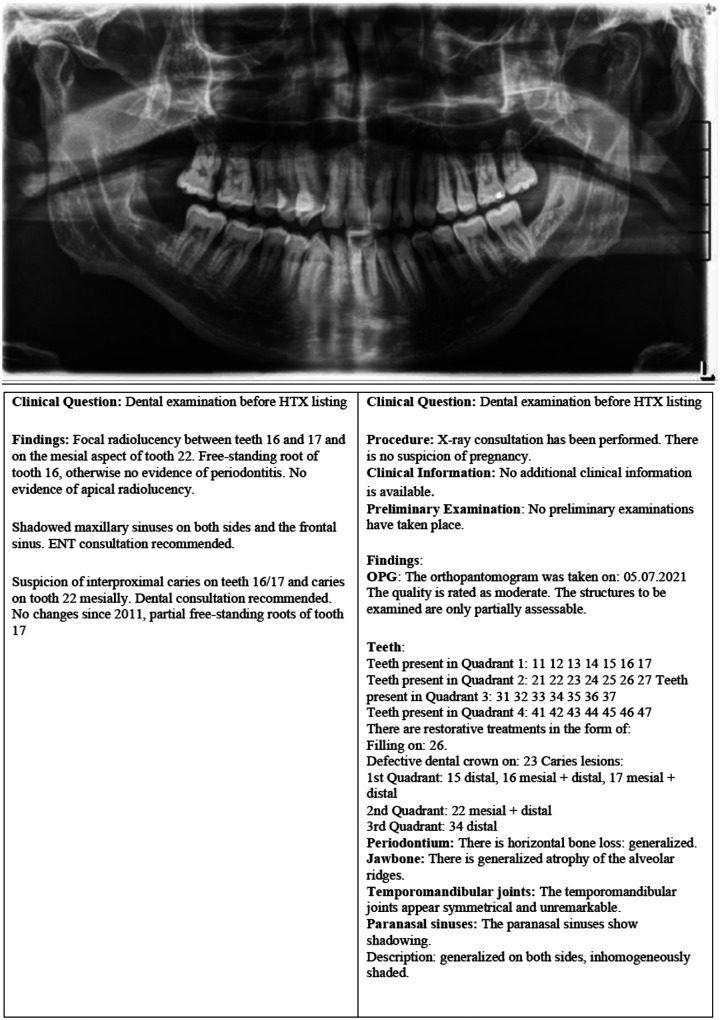
DPI of a patient for dental examination before heart transplantation (HTX) listing with correlating free-text reports as well as the structured report.

All SRs were retrospectively generated by a board-certified dentist using the Smart Radiology online software (Smart Reporting GmbH, Munich, Germany). The template featured a clickable decision tree with dropdown menus, yes/no questions, and free-text fields. By selecting relevant options, the software automatically generated structured sentences from predefined text modules, while free-text fields allowed for additional input when necessary. An example of an DPI with corresponding NR and SR is shown in Figure 2. The final reports were then exported and stored (23).

#### 2.2.4 Evaluation of reports

All SRs and NRs in this study were evaluated by two additional board-certified dentists using a questionnaire specifically designed to assess the quality of the reports and their utility for referring physicians. The questionnaire (Figure 1) was developed by the dentist who also created the SRs and corresponding template, based on prior studies (23, 24, 26, 27). The questionnaire was not formally validated or pilot-tested, and therefore its psychometric robustness and reproducibility must be interpreted with caution. The evaluation criteria included whether the referring physician's clinical question was addressed, whether the report provided sufficient information for determining the clinical course and therapy, and whether the data extracted from the DPIs were adequate for the chosen therapy. Additionally, missing relevant information was documented.

Missing key features included essential findings such as dental anomalies, restorations, carious lesions, periodontal structures, apical changes, jawbone condition, temporomandibular joints, and paranasal sinuses. Both positive and negative findings were assessed to ensure completeness.

Evaluators also rated how easily information could be extracted, whether the report structure facilitated this process, and their trust in the information. Linguistic and overall report quality were scored on a six-point Likert scale (1 = poor/no trust; 6 = excellent/full trust).

After anonymization and randomization, two experienced dentists independently reviewed 100 reports (50 NRs and 50 SRs) corresponding to 50 dental panoramic images. For each image, each evaluator assessed both report types in separate reading instances; each report was presented together with the corresponding radiograph and the clinical question. The questionnaire was completed immediately after each evaluation. Although the reports were randomized, this design meant that both formats for the same case were ultimately seen by the same evaluators, which may introduce recall bias. Disagreements between evaluators were not resolved through consensus; instead, each rating was analyzed independently.

### 2.3 Statistical methods

Statistical analyses were performed using proprietary statistical software (IBM SPSS Statistics Version 25, Armonk, New York, NY, USA). For questions 1–6, the McNemar test was used to compare binomial data and determine statistical significance. For paired data related to questions 7–9, the Wilcoxon signed-rank test was applied, with a confidence interval (CI) of 95%. Mean Likert-scale ratings for questions 7–9 were calculated by combining the results of both evaluators. Inter-rater agreement was assessed using Cohen's kappa coefficient. The significance level was set at *α* = 0.05 (24). All tests were two-sided, and no dichotomization was applied.

## 3 Results

Both dentists retrospectively completed 50 questionnaires each for the NRs and 50 for the generated SRs corresponding to the 50 randomly selected DPIs. The interrater agreement between the two evaluators was substantial, with a Cohen's Kappa of 0.786. The results are presented as percentages or means with corresponding CI.

All SRs (100%) answered the referring physician's clinical question, compared to 42% of the NRs (*p* < 0.05). Similarly, all SRs (100%) facilitated decision-making regarding subsequent clinical management, whereas only 29% of NRs did so (*p* < 0.05). The information provided was sufficient for the chosen treatment in 100% of the SRs, compared to 27% of the NRs (*p* < 0.05).

Moreover, at least one critical key feature was missing in only 3% of SRs, whereas this was the case in 96% of NRs (*p* < 0.05). The extraction of information was rated as “easy/quick” in 100% of the SRs and in 52% of the NRs (*p* < 0.05). For NRs, information extraction was considered “with effort” in 45% of cases and “very time-consuming” in 3%. The structure of the report was perceived as helpful for extracting information in 100% of the SRs, compared to 39% of the NRs (*p* < 0.05).

The evaluating dentists expressed significantly greater trust in the information provided by SRs, with a mean score of 5.97 (CI: 5.94–6.00) on the Likert scale, compared to 4.27 (CI: 4.14–4.40) for NRs (*p* < 0.05). SRs also received significantly higher ratings for linguistic quality, with a mean score of 6.00 on the Likert scale, compared to 5.42 (CI: 5.28–5.56) for NRs (*p* < 0.05). The overall report quality was significantly improved through structured reporting, with SRs receiving a mean score of 5.97 (CI: 5.94–6.00), compared to 4.53 (CI: 4.41–4.65) for NRs (*p* < 0.05).

**Figure 3:**
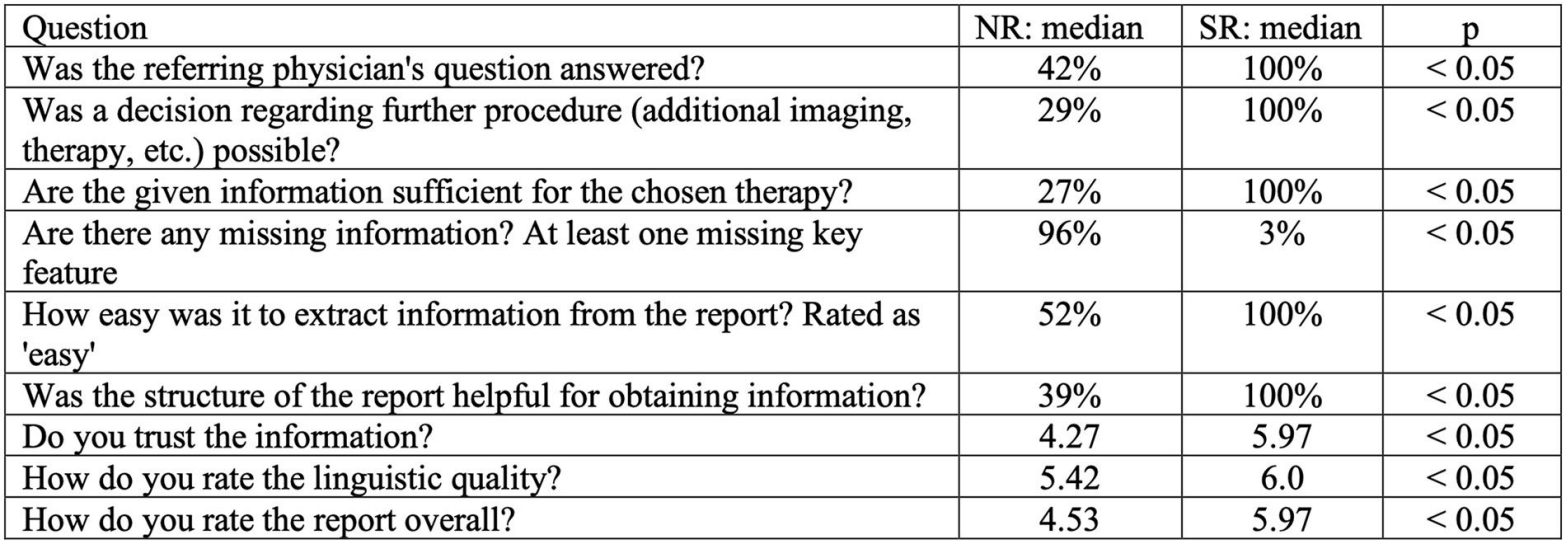
Results are presented as percentages for questions 1–6 and as mean values for questions 7–9 after statistical analysis using IBM SPSS statistics.

Cohen's Kappa values for each question were as follows: question 1 resulted in 0.834; question 2 in 0.576; question 3 in 0.879; question 4 in 0.910; question 5 in 0.823; question 6 in 0.835; question 7 in 0.903; question 8 in 0.467; and question 9 in 0.844. An overview of the results for the questionnaire after statistical analysis can be seen in Figure 3. It should be noted that the differences observed in report completeness, clarity, and clinical utility may partly reflect disparities in professional training and familiarity with oral and maxillofacial imaging, rather than representing inherent advantages of SR over NR.

## 4 Discussion

This study represents, to our knowledge, the first investigation evaluating the benefits of SR for DPIs and oral and maxillofacial imaging in a hospital-based radiology setting. A total of 50 DPIs with corresponding NRs were retrospectively and randomly selected from the clinical information system, covering cases from December 2008 to February 2022.

### 4.1 Methods

This retrospective clinical data analysis leveraged pre-existing DPIs and NRs, allowing a timely and resource-efficient evaluation of reporting quality under real-world conditions. While retrospective designs limit control over data quality, they avoid potential Hawthorne effects that might occur in prospective studies, where radiologists could consciously or unconsciously modify their reporting behavior (28).

A key constraint of this design was the inability to measure NR-time, which is crucial for assessing efficiency. In addition, the single-center setting restricted the spectrum of clinical indications, as most DPIs were obtained to exclude dental foci prior to transplantation or oncologic treatments. Consequently, the present work should be regarded as a preliminary study that primarily reflects the realities of a hospital environment rather than routine dental practice.

### 4.2 Results

In recent years, numerous publications have demonstrated that SR can improve report quality, interdisciplinary communication, and perceived clinical utility in various radiological subspecialties (23, 24, 26, 27). Oral and maxillofacial imaging, however, has largely been excluded from these analyses. This study therefore aimed to explore whether the advantages of SR could be transferred to DPI, a standard diagnostic modality in dentistry.

SRs significantly outperformed NRs in addressing clinical questions, supporting clinical decision-making, and providing the information deemed necessary for therapeutic planning. They contained fewer omissions of key features and enabled substantially faster and easier information extraction for the evaluators. These findings are consistent with the conceptual strengths of SR: the decision-tree-based template guided the reporting clinician systematically through all relevant anatomical structures and clinical aspects, thereby reducing the risk of overlooking important findings and enhancing clarity and comprehensiveness. Standardized text modules also contributed to higher linguistic and overall quality compared with NRs, which are typically generated under time pressure and may be more susceptible to ambiguity.

The decision-tree approach inherent in SR templates reduced the likelihood of missing essential information, as reporting clinicians were systematically guided through all critical aspects of the report. This increased completeness naturally improved responses to clinical inquiries and supported further diagnostic and therapeutic planning. Templates also serve as valuable tools for medical students and less experienced radiologists, providing a structured framework for report content and organization (20, 29–31). However, adapting to new technologies can be challenging for senior radiologists, potentially leading to temporary reductions in productivity as established workflows are disrupted (32).

The standardized structure of SRs promotes faster information retrieval for referring clinicians, contributing to time savings, a benefit supported by prior research (26, 33, 34). However, the time required for generating SRs vs. NRs remains a topic of debate, with inconsistent findings in other imaging modalities. This aspect could not be evaluated in the current study due to its retrospective design (22, 35, 36).

In this context, the particularly pronounced differences observed for questions 2, 3, and 6 warrant further consideration. Questions 2 and 3 addressed whether the report provided sufficient information for clinical decision-making and for selecting an appropriate therapy. These items integrate both the completeness of reported findings and the evaluators’ clinical expectations and may therefore be especially sensitive to differences in perspective between dentists and medical radiologists. By contrast, question 6 explicitly focused on whether the report structure facilitated information extraction, directly capturing a core strength of structured reporting. It is thus not surprising that SR showed the largest advantage in this domain. Together, these findings suggest that the added value of SR may be most evident where clinical decision support and formal report organization are closely intertwined.

The linguistic and overall quality of SRs was consistently superior to that of NRs. Templates ensure the use of pre-validated, standardized text modules, leading to reports that are clear, precise, and professionally formulated. In contrast, time pressures in clinical routines often result in ambiguous or unclear language in NRs, which can cause confusion, require additional clarification from radiologists, or, in the worst cases, lead to errors in patient care. The higher quality of SRs, attributed to improved accuracy, clarity, and completeness, has been corroborated by multiple studies in recent years (25, 27, 35–40).

### 4.3 Limitations

This study has several limitations. First, the templates and SRs were generated exclusively by a single dentist, whereas the NRs were authored by multiple radiologists with different backgrounds. As a result, the higher linguistic quality of SRs might reflect the individual skills and specific expertise of this dentist rather than a generalized benefit applicable to all reporting clinicians. The free-text reports were produced over a long period (2008–2022) by radiologists of varying ages, training backgrounds, and levels of experience, which inevitably led to heterogeneity in style and wording. In this context, the assessment of linguistic quality is particularly susceptible to bias, as it reflects not only differences between SR and NR formats but also this underlying variability among NR authors. Moreover, the interpretation of linguistic quality is likely influenced by the evaluators’ own professional and linguistic preferences as dentists. It should be noted that, in the daily clinical practice of a medical radiologist, dental panoramic imaging is relatively uncommon, whereas for a dentist the interpretation of a dental panoramic image is an integral part of routine diagnostics. Consequently, the positive results associated with SRs cannot be generalized to all reporting physicians. Moreover, neither the author of the structured reports nor the evaluators were oral and maxillofacial radiologists with formal subspecialty training in dentomaxillofacial imaging. The intention of this study is therefore not to suggest that medical radiologists should not engage in oral and maxillofacial imaging, but rather to illustrate how appropriate training and the use of structured templates, regardless of specialty background, may help to improve consistency and transparency in dental panoramic image reporting.

Additionally, the SRs were created by one dentist and evaluated by two others, who likely shared similar expectations regarding the level of detail and structure considered relevant in DPI reporting. Differences in reporting conventions between dentists and radiologists may therefore have contributed to lower ratings for NRs. This may also reflect that the clinical questions prompting DPI acquisition in a hospital environment differ from those commonly encountered in dental practice, which naturally influences the type of information emphasized in the report. Moreover, the evaluators were not blinded to the report format and assessed both SRs and NRs for the same cases, which may have introduced recall bias and further favored SR. While the study confirmed SR's advantages in clarity and completeness, subjective biases inherent to the retrospective design must be acknowledged as a limitation.

Another limitation is the small number of evaluators, as only two experienced dentists assessed the reports. Furthermore, the questionnaire used to evaluate report quality was specifically developed for this study and was neither formally validated nor pilot-tested. Moderate inter-rater agreement for some items suggests that certain questions may have allowed for subjective interpretation, and the instrument's ability to comprehensively and reliably capture “report quality” is therefore limited. In addition, the Likert-scale items applied in this study are ordinal in nature. For simplicity and in line with common practice in the radiological literature, we reported means and confidence intervals; however, medians might have provided a more conservative description of the data. This is particularly relevant for question 8, for which inter-rater agreement was only moderate, and the corresponding results should therefore be interpreted with caution. However, this study aligns with several previously published investigations that explored the potential benefits of SR in radiology (23–25, 27). To mitigate some of these limitations, both evaluators possessed substantial expertise in DPI interpretation and were tasked with independently and objectively completing the evaluation without prior knowledge of expected outcomes.

Additionally, the SRs and NRs were not created by the same individuals due to the retrospective study design. The dentist generating the SRs may have worked more diligently and attentively in the context of the study than the radiologists who created the NRs under time pressure in their routine clinical workflow. This design also did not allow for evaluating the impact of SRs on reporting time or productivity; the study could only demonstrate that SRs facilitated faster and more efficient information extraction for the evaluators.

Another limitation is that there may be an observed training effect in questions 5 and 6 (Figure 1). This suggests that repeated evaluation of similarly structured findings naturally favors SR, which aligns with its intended purpose and supports its implementation. However, this does not necessarily imply that a third reader, without prior exposure to previous SRs, would find an SR equally intuitive or helpful, as a well-written NR may, in some cases, provide superior clarity and diagnostic value.

The study also faced limitations regarding the dataset. The radiological information system at the Department of Radiology predominantly contained DPIs used to assess dental conditions prior to planned radiotherapy, chemotherapy, or immunosuppressive treatments. A larger and more diverse dataset could be analyzed in future studies in order to validate the findings of this study and to explore the performance of SR across a broader spectrum of clinical indications.

## 5 Conclusion

This study provides preliminary evidence that SR offers relevant advantages over NR for DPI, particularly regarding completeness, readability, and the perceived support for clinical decision-making and interdisciplinary communication. Within the specific hospital-based setting examined here, SR improved the systematic documentation of key findings and facilitated faster information extraction for the evaluators.

However, these results must be interpreted with caution. The unequally trained authorship of SRs and NRs, the exclusive involvement of dentists as evaluators, and the use of a non-validated questionnaire limit the generalizability of the findings and preclude definitive conclusions about the inherent superiority of SR over high-quality narrative reports in other settings. Rather than a final verdict, this work should be understood as an exploratory step that highlights the potential of SR to standardize and structure DPI reporting.

Future research should build on these preliminary observations using multicenter, prospective designs with standardized report authorship across formats, validated evaluation instruments, and the inclusion of radiologists and clinical end users as evaluators. Such studies should also assess reporting time, cost-effectiveness, diagnostic accuracy, and patient outcomes, and may explore the integration of AI-assisted SR generation. In this context, specialty-specific templates and hybrid models combining structured elements with free-text flexibility are likely to be key for successful and broadly acceptable implementation.

## Data Availability

The raw data supporting the conclusions of this article will be made available by the authors, without undue reservation.
